# The SUPERFAMILY 1.75 database in 2014: a doubling of data

**DOI:** 10.1093/nar/gku1041

**Published:** 2014-11-20

**Authors:** Matt E. Oates, Jonathan Stahlhacke, Dimitrios V. Vavoulis, Ben Smithers, Owen J.L. Rackham, Adam J. Sardar, Jan Zaucha, Natalie Thurlby, Hai Fang, Julian Gough

**Affiliations:** 1 Computer Science, University of Bristol, Bristol, BS8 1UB, UK; 2 Medical Research Council Clinical Sciences Centre, Faculty of Medicine, Imperial College London, Hammersmith Hospital, London, UK; 3 e-Therapeutics plc,17 Blenheim Office Park, Long Hanborough, Oxfordshire, OX29 8LN, UK; 4 Bristol Centre for Complexity Sciences, University of Bristol, Bristol, UK

## Abstract

We present updates to the SUPERFAMILY 1.75 (http://supfam.org) online resource and protein sequence collection. The hidden Markov model library that provides sequence homology to SCOP structural domains remains unchanged at version 1.75. In the last 4 years SUPERFAMILY has more than doubled its holding of curated complete proteomes over all cellular life, from 1400 proteomes reported previously in 2010 up to 3258 at present. Outside of the main sequence collection, SUPERFAMILY continues to provide domain annotation for sequences provided by other resources such as: UniProt, Ensembl, PDB, much of JGI Phytozome and selected subcollections of NCBI RefSeq. Despite this growth in data volume, SUPERFAMILY now provides users with an expanded and daily updated phylogenetic tree of life (sTOL). This tree is built with genomic-scale domain annotation data as before, but constantly updated when new species are introduced to the sequence library. Our Gene Ontology and other functional and phenotypic annotations previously reported have stood up to critical assessment by the function prediction community. We have now introduced these data in an integrated manner online at the level of an individual sequence, and—in the case of whole genomes—with enrichment analysis against a taxonomically defined background.

## INTRODUCTION

SUPERFAMILY (1) is both a database and website resource available freely to the public. Predicting the presence of protein domains of known structure in amino acid sequence is the main focus of the resource, but over time additional annotations and features for functional and phylogenetic analysis have been introduced. The database is built around a library of 15 438 expert-curated hidden Markov models (HMM) representing all protein domains of known structure. The classification of these domains is taken from the Structural Classification of Protein (SCOP) database (2). SCOP classifies protein domains into a hierarchy according to similarity in structure, at increasing levels of similarity and evolutionary relationship including: *Class*, *Fold*, *Superfamily* and *Family* levels. The SUPERFAMILY database focuses on the *Superfamily* level, but additionally provides protein domain assignments at the *Family* level (3). Two domains are strictly grouped into the same *Superfamily* if there is structural evidence for a common ancestor (4). In general, the *Family* level SCOP annotation has also been found to be functionally consistent (5), and has a closer evolutionary relationship often directly observable at the level of amino acid sequence. For these reasons, inclusion of a domain at both the *Superfamily* and *Family* level within a species can be thought of as evolutionary characters. The genomic inclusion of an individual domain annotation and in combination with other domains is the basis of our daily updated phylogenetic tree of life (6).

The SUPERFAMILY website offers a variety of methods to analyse whole proteins and domains. A keyword search is available from all pages on the site, and sequences can be directly annotated against the HMM library from the ‘Sequence search’ page. At the genomic level the user can investigate under- and overrepresentation of domains (4), view phylogenetic trees (6), plot domain architectures and networks (7) and examine the distribution of domain superfamilies or families across the tree of life (8). The dcGO resource (9) has undergone continued development providing improved ontological annotations for SUPERFAMILY domains and their combinations, more on this is discussed below.

Here, we primarily wish to describe extensive expansion of the curated sequence collection provided through SUPERFAMILY, as well as introduce some new features to the SUPERFAMILY 1.75 website since last publication (10). In the next section we describe in detail how the complete proteomes sequence library has expanded; followed by summarizing the technological improvements in SUPERFAMILY 1.75 since October 2010. In the last section we discuss related spin-off sister projects that have formed around the core features that SUPERFAMILY provides, as well as showcase interesting developments that SUPERFAMILY has facilitated since the release of 1.75. Finally, discussion is given on the future direction of SUPERFAMILY with respect to the upcoming SCOP2 resource (11).

## SUMMARY OF UPDATES

### Expansion of the complete proteome sequence collection

The version number of SUPERFAMILY resource is tied to the SCOP version of the HMM library, and does not reflect changes to the collection of sequences we provide domain annotations for. In Table 1 we can see a summary of the expansion of proteomes available grouped by domain of life; this table in combination with Table 2 demonstrates that despite the doubling of sequence data we have not seen a dramatic reduction in the ability of our HMM library to assign domain homology. In Bacteria, for example, there is only a 0.81% drop in sequences with assignment to at least one SCOP Superfamily, and only a 0.43% drop in total amino acid sequence coverage despite a doubling in sequence data since the initial release. Eukarya have infact improved in amino acid coverage by ∼1%, while Archaea have lost ∼1%. In general, the number of completely unannotated sequences has increased for whole proteomes, but the more global ability to recognize and annotate domains remains relatively constant looking at the whole of UniProt.

**Table 1.** A summary of the proteomes included in SUPERFAMILY 1.75 comparing the currently available database with the initial release

	SUPERFAMILY 1.75 in 2010	SUPERFAMILY 1.75 in 2014
Eukaryota	341	498
Archaea	87	165
Bacteria	1177	2595
Viruses	NA	5239
Plasmids	2354	4573
Metagenomes	118	121
UniProt (Complete Proteomes)	NA	5255

**Table 2.** A summary of the HMM sequence coverage in SUPERFAMILY 1.75 comparing the currently available database with the initial release

	Proteins with assignments (%)		Amino acid coverage (%)	
	2010	2014	2010	2014
Eukarya	59.11	56.8	38.9	39.91
Archaea	65.13	62.9	61.67	60.5
Bacteria	68.08	67.27	63.4	62.97
Viruses	NA	63.0	NA	25.52
Plasmids	47.0	48.79	47.0	48.22
Metagenomes	51.47	57.67	54.1	60.48
Protein Data Bank	NA	89.94	NA	89.11
UniProt (All Proteins)	64.0	64.73	56.0	58.78

**Figure 1.**
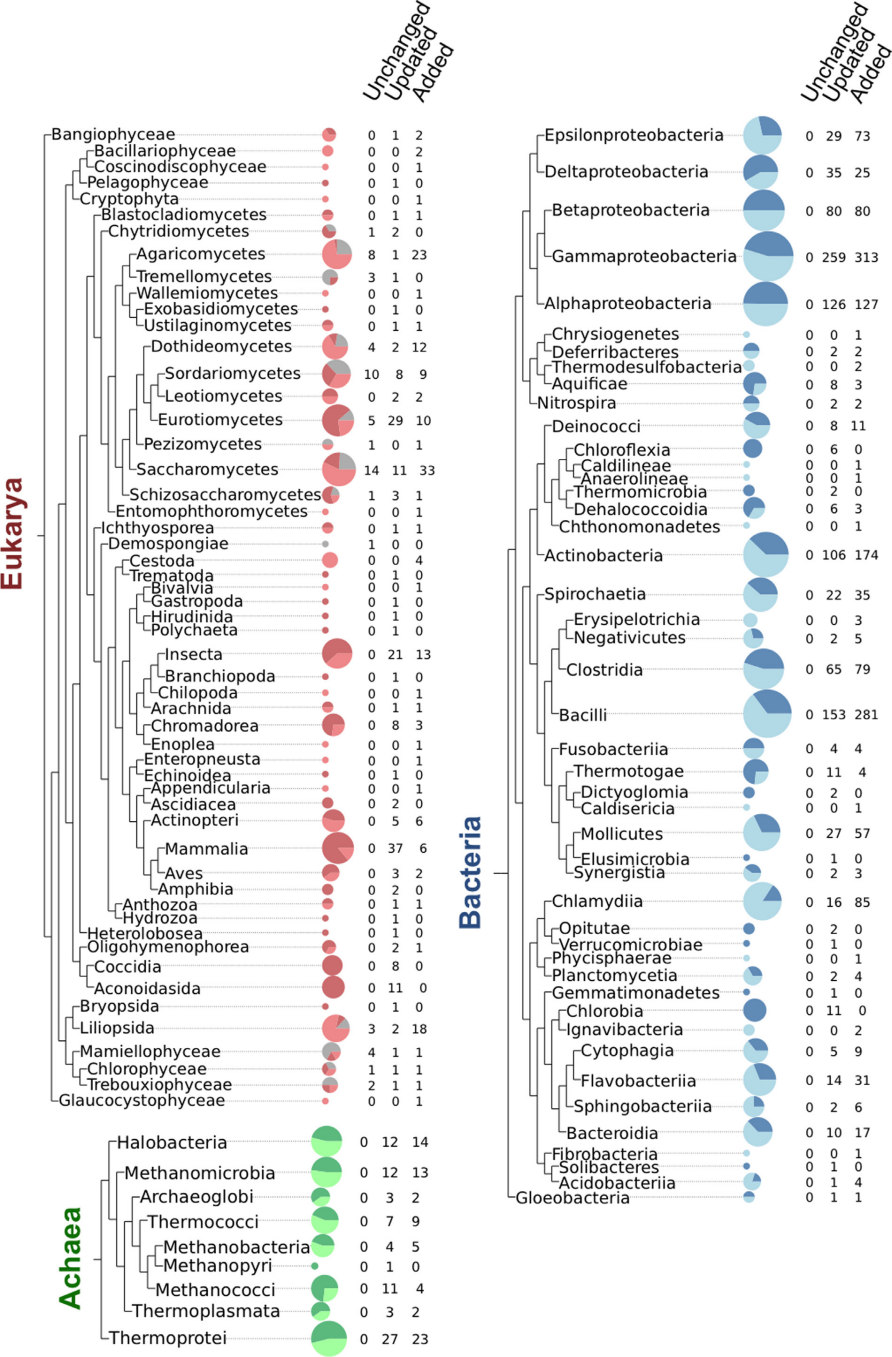
Summary of all genome updates and additions at the level of taxonomic Class since the release of SUPERFAMILY 1.75. Eukarya in red, Archaea in green and Bacteria in blue. The size of each pie chart is log scaled based on the number of proteomes within each Class. Light colouration is the proportion of genomes that have been added to the database within a Class, and the dark colouration represents updated genomes. The grey colouring seen in Eukarya represents the relatively few genomes to not have been altered since the release of 1.75.

In Figure 1 we present SUPERFAMILY's curated complete proteomes coverage over the tree of all sequenced life collapsed to the rank of Class as defined by the NCBI taxonomy. This does not include all of the species-specific annotations we serve as part of UniProt proteomes collection, but does include collections from NCBI RefSeq (as of 13 August 2014) (12) and Ensembl (release 76) (13) as well as hundreds of complete proteomes we have acquired from various individual sources (upon publication). Of special note is that SUPERFAMILY now provides assignments to the latest assembly of Human GRCh38 thanks to its recent inclusion in Ensembl. The new Human assembly has 61% of sequences with at least one domain annotation, and 44% of all amino acids being annotated with SCOP domains.

Only 64 complete eukaryotic proteomes have remained unchanged since the release of SUPERFAMILY 1.75. The proteome sequence collection represents 1714 species and 1544 strains, totalling coverage of 3258 complete cellular genomes. Greater focus has been given to proteomes forming mobile genetic elements too, with 5238 complete viral proteomes made available as the unison of UniProt (14) and NCBI viral (15) collections, and 4523 plasmid proteomes from NCBI RefSeq plasmids (12).

**Figure 2.**
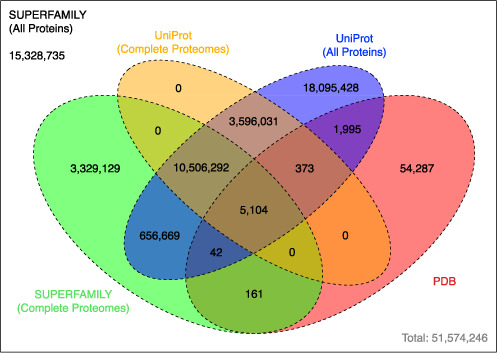
This Venn diagram demonstrates the extent to which the sequence space of the SUPERFAMILY proteome collection is not covered by the PDB and UniProt. Each value in the diagram describes the number of distinct (collapsed to 100% sequence identity) amino acid sequences in each sequence collection.

In total 1376 proteomes have been updated to a new version with 1818 completely new proteomes added since the original 1.75 release: 679 representing completely new species (181 Eukarya, 450 Bacteria, 48 Archaea), and 1139 strains of existing species (30 Eukarya, 1076 Bacteria, 33 Archaea). Of special note, 40 new *Viridiplantae* proteomes have been added since the last release, bringing the total number to 59. This extends the represented species of other resources, such as Phytozome (version 10.0.4) (16), that provided 48 of the proteomes included in SUPERFAMILY. At the level of individual sequences SUPERFAMILY now contains 34 222 445 linked sequence objects in the complete proteomes collection (including species with draft assemblies), with a total of 111 392 143 linked protein sequences covering other sources, such as UniProt. For a more detailed view of the sequence space interface between UniProt, SUPERFAMILY and known structures from the Protein Data Bank (PDB; as of 18 September 2014) please see Figure 2.

As we can see from Figure 2, the total number of distinct protein sequences found only in UniProt (3 596 031) and SUPERFAMILY (3 329 129) complete proteome collections is similar. The additional diversity of taxonomic coverage is similarly represented between the databases with: six additional classes covered in UniProt, and nine in the SUPERFAMILY complete proteome collections. However, there are 1195 additional proteomes found only in UniProt, and 356 found only in SUPERFAMILY. Although the total number of extra proteomes is less in SUPERFAMILY this represents a similar level of additional sequence and taxonomic diversity. As Supplementary Material we have provided lists of specific species that might aid better representation of taxa in both SUPERFAMILY and UniProt collections. It is worth noting that all of these additional proteomes are included in SUPERFAMILY as part of our domain assignments to UniProt (http://supfam.org/SUPERFAMILY/cgi-bin/gen_list.cgi?genome=up;subgenome=all), but are currently not available for phylogenetic analysis via the web interface.

In Figure 1 we can see that since the release of SUPERFAMILY 1.75 many new proteomes have been produced and incorporated. In many cases the number of proteomes in a class has doubled or more than doubled, with eukaryotic proteomes having been produced to address previously limited taxonomic coverage. Of the 267 NCBI defined taxonomic classes 109 now have at least one proteome representing that clade, with 22 new classes having representation since the release of SUPERFAMILY 1.75. Specifically 12 new eukaryotic and 10 bacterial classes are defined. Many classes are highly overrepresented in regard to taxonomic representation, such as *Gammaproteobacteria*, because they are of significant scientific and medical importance, due to their role in disease. To this end we have begun to address the issues of proteome curation for selecting representatives of a given clade for evolutionary analysis, please see discussion of the Proteome Quality Index (PQI) resource below.

### Daily updated phylogenetic tree

**Figure 3.**
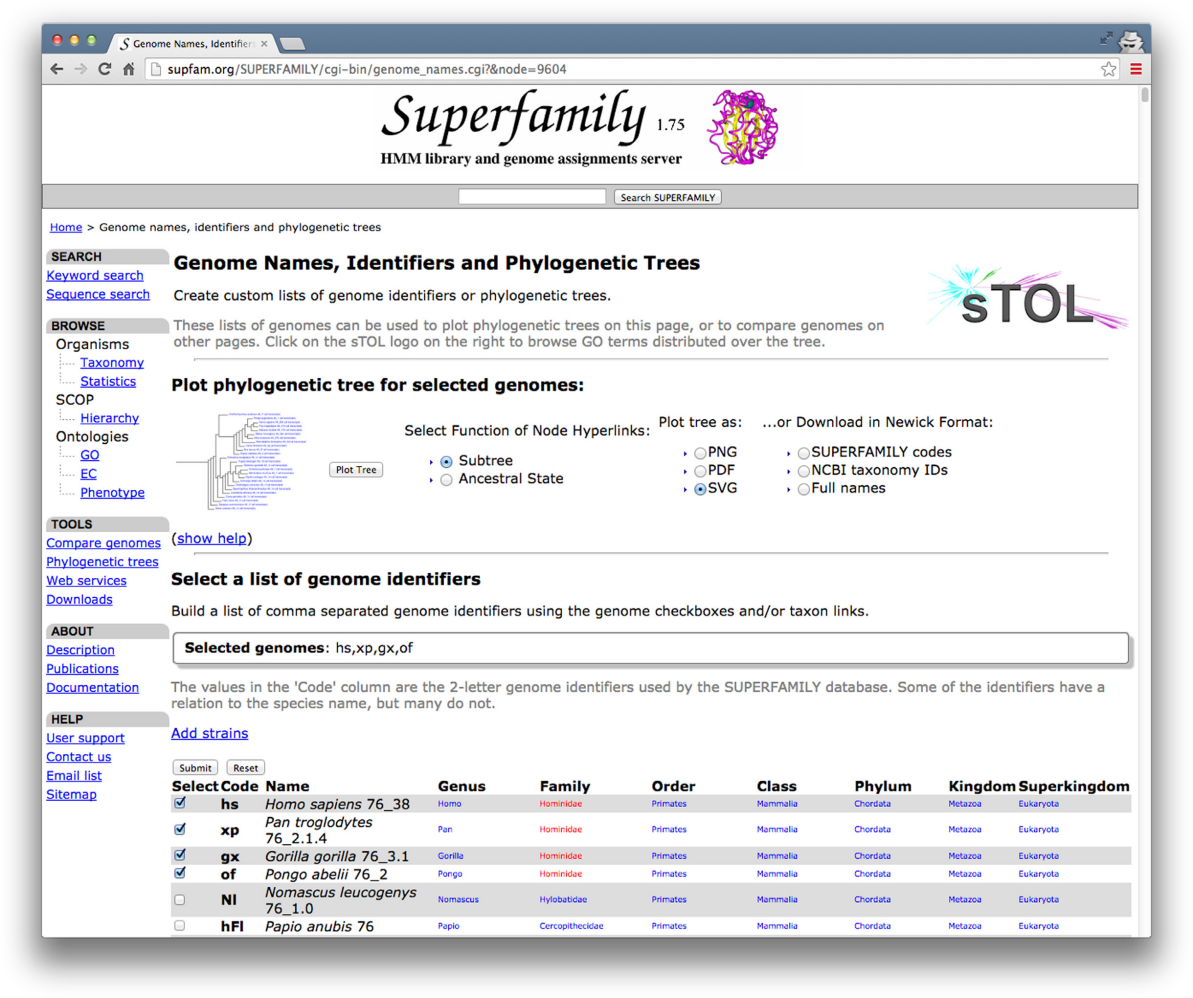
How to create your own phylogenetic trees that are built daily against the most recent updates to the SUPERFAMILY sequence collection. In this example the family *Hominidae* has been selected from the table and links to phylogenetic resources provided by the sTOL method given at the top of the page. A user may also select individual species of interest and create trees annotated by domain inclusion directly from domain summary pages.

**Figure 4.**
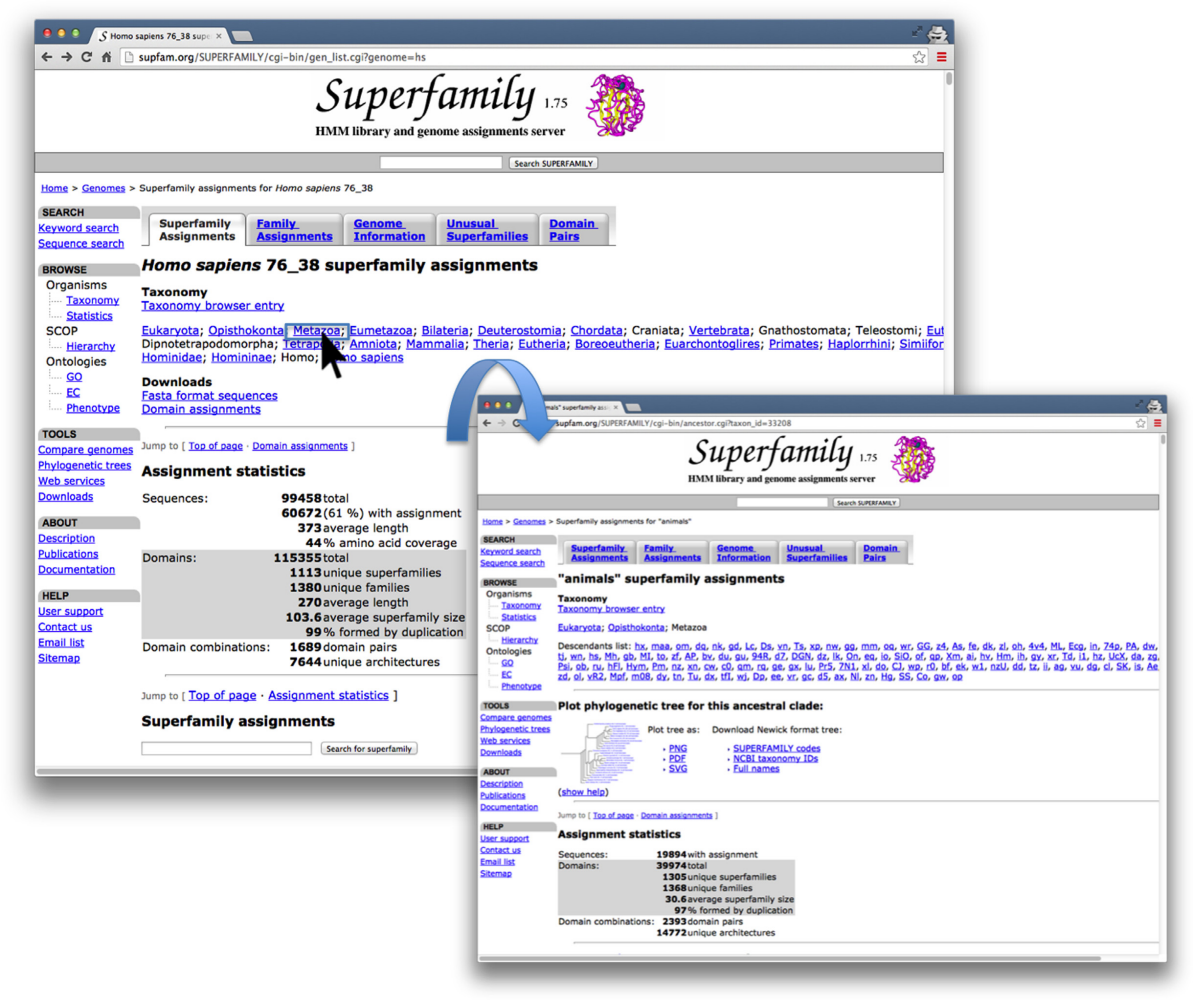
In this figure we demonstrate viewing the ancestral domain content for the last common ancestor to all *Metazoa*, linked from the summary of domain assignments for *Homo sapiens*. From the main SUPERFAMILY assignments page for a proteome (accessible from the Taxonomy page under Browse on the side menu) a user can view reconstructed ancestral states for any common ancestor as long as the clade has sufficient whole proteome data.

SUPERFAMILY 1.75 introduced the printing of annotated phylogenetic trees using TreeVector (17) to investigate domain inclusion within a clade. The sTOL (6) methodology was produced so that the data behind this functionality could be updated constantly within a day of data being added to the sequence collection. In Figure 3 we demonstrate how to view a clade of your choice from the latest sTOL phylogenetic tree now available at http://supfam.org/SUPERFAMILY/sTOL. For all Eukarya SUPERFAMILY now makes it possible to view predictions for ancestral proteome domain inclusion using Dollo parsimony (18), as convergent evolution of domain combinations is rare (19). In Figure 4 you can see how to view the plausible domain content of the last common ancestor to all extant *Metazoa*. This page is easily reachable from clicking a named link in the full taxonomic classification of any eukaryotic species on the main genome assignment page.

### Improved integration of Gene Ontology annotation and prediction through dcGO

The dcGO is a comprehensive database for annotating domains using a panel of ontologies. This resource has proven useful in achieving a domain-centric functional understanding of protein sequences, as seen in the Critical Assessment of Function Annotation competition for computational protein function prediction (20).

A top-ten list of highly specific Gene Ontology (GO) terms is now provided at the top of each per-sequence SUPERFAMILY page. This links out to the complete and rigorous set of annotations provided by dcGO for further analysis, including the use of dcGO Predictor (21) that provides broader functional and phenotypic prediction. In addition to individual sequences, GO terms for a whole proteome can be identified as unusual relative to a background. This background can be customized, for example, choosing a closely related species or group of species within the same *Genus* or *Family*. The list of unusual GO terms can be used to implicate what functions were gained or lost in this proteome.

## DISCUSSION OF COMMUNITY ENGAGEMENT AND FACILITATED ANALYSIS

### PQI

The PQI (http://www.pqi-list.org) is an initiative effort producing a customer review style 1–5 star rating of proteome quality for fully assembled genomes. The rating is based on a combination of different scoring metrics. All completely sequenced genomes in SUPERFAMILY have been rated. Ratings of 3-stars or more are indicative of proteomes considered suitable for general use, and 2-stars or less considered for speculative use or when no coverage exists otherwise in a clade. This quality rating gives a feedback to our internal curators, but more importantly to our end-users (and genome providers) for what kind of data they are consuming or providing.

### Genome3D

Genome3D (22) is a collaborative project aiming to provide consensus prediction of protein structure using various sources of structured domain annotations, and 3D models. An additional goal of Genome3D is to integrate CATH (23) and SCOP domain classifications and rate the quality of agreement between these two systems. As a Genome3D partner, SUPERFAMILY domain annotations are included as both HMM assignments, and 3D models. Full 3D models were created from SUPERFAMILY HMM assignments using Modeller (24) and a template structure from the nearest PDB structure as defined by SCOP. Results of structural domain assignment and 3D modelling for all sequences for several model organisms—including human—can be found online (http://genome3d.eu).

### D^2^P^2^ the database of disordered protein predictions

D^2^P^2^ (25) has been produced as an extension of the SUPERFAMILY database schema and provides complementary annotation of predicted disordered protein state. Any user of SUPERFAMILY wishing to investigate the role of disordered protein regions in relation to SCOP structural domains may wish to use this sister resource. It is anticipated that D^2^P^2^ will be updated to use the latest SUPERFAMILY complete genome sequence collection within the year.

## FUTURE DIRECTIONS

SCOP2 (11) represents a new methodology of protein structure classification building on the basic principles of SCOP 1.75. However, there remains a disparity at the time of writing between PDB coverage of SCOP2 and SCOP 1.75 resources. During this transitional period a new web resource SUPERFAMILY 2 is being developed to modernize the SUPERFAMILY database and website, improving the user experience and putting in place the required infrastructure for anticipated future requirements.

The SUPERFAMILY resource although primarily aimed at bringing user facing value also provides a robust server side pipeline for processing of whole genome protein annotations, in a timely manner. Analysis produced in the creation of sister resources, such as D^2^P^2^, will be, in part, included in SUPERFAMILY 2, allowing for additional molecular annotations to be produced as and when sequences are published.

## SUPPLEMENTARY DATA

Supplementary Data are available at NAR Online.
